# Epidémiologie des PFA et les performances du système de surveillance en Mauritanie de 2008 à 2012

**DOI:** 10.11604/pamj.2014.18.305.3362

**Published:** 2014-08-15

**Authors:** Jean Gérard Tatou Doumtsop, Ishagh Khalef, Med Lemine Brahim Diakite, Naouri Boubker

**Affiliations:** 1 Bureau OMS, Nouakchott, Mauritanie; 2 Ministère de la Santé Publique, Cameroun; 3 Ministère de la Santé Publique, Mauritanie; 4 Groupe de support inter-Pays pour l'Afrique de l'Ouest, OMS, Ouagadougou

**Keywords:** Poliomyélite, PFA, épidémiologie, surveillance, éradication, Poliomyelitis, AFP, epidemiology, surveillance, eradication

## Abstract

**Introduction:**

La Commission Régionale de Certification de l'Eradication de la poliomyélite pour l'Afrique(CRCA) en session à Brazzaville du 8 au 10 octobre 2007 avait déclaré la Mauritanie « libérée de la poliomyélite ».

**Objectif:**

Décrire l’épidémiologie des PFA (Paralysies flasques aigues) et évaluer les indicateurs de performance du système de surveillance pour la période de 2008 à 2012 ayant suivi cette déclaration.

**Méthodes:**

Les données du service de surveillance épidémiologique ont été nettoyées et analysées à l'aide du logiciel Epi-infoversion 3.4.3 (CDC Atlanta).

**Résultats:**

319 cas de PFA ont été notifié soit une incidence moyenne de 4.61/100000 enfants de moins de 15 ans par an. La distribution des cas cumulés par mois montre une importante notification des cas PFA pendant la période de Février à Juillet et à la suite de l’épidémie de 2009 alors que l'incidence des cas confirmés a été plus importante entre Novembre et Février. L’âge moyen était de 4ans (E.T. ±4ans) et 77.4% avaient un âge = 5ans. 18 cas de PFA ont été confirmés poliovirus sauvage(PVS) dont 6 en 2009 et 12 en 2010 et tous d'importation. L’âge moyen était de 3.4 ans (E.T ±2.6 ans). 44,4% étaient des filles et 55.5% garçons. Cette proportion était proche de celle des PFA non polio (45.1% versus 54.9%). 61% avaient reçu au plus une dose de vaccin polio orale (VPO) pour les cas de PFA polio contre 7.4% pour les PFA non polio. Aucune discrimination de genre n'a été observé sur la population des PFA ayant reçu une dose au plus (ratio-sexe= 16/17=0.94). La fièvre était présente pour 90%des cas de PFA non polio contre 85% pour les cas PVS. Cette fièvre à progresser en 3 jours pour tous les cas de PVS et pour 82,7% des cas de PFA non polio. Le taux d'hospitalisation était de 13.6% pour les cas de PFA non polio contre 89% pour les PFA polio. Dans tous les deux groupes, les membres de prédilection étaient d'abord l'un des 2 membres inférieurs de façon alternative (46.8%), ensuite les deux membres inférieurs à la fois (22.2%). En troisième lieu, un membre supérieur et un membre inférieur de façon asymétrique (5.3%) et en quatrième lieu, tous les 4 membres (4.6%). Le diagnostic final des cas de PFA non polio n’était pas rapporté pendant toute la période de l’étude, cependant, 2 cas ont été classés compatible et un cas de paralysie associée à la vaccination contre la polio a été rapporté en 2012 chez un enfant féminin de 3 ans ayant reçu sa première dose de VPO. En ce qui concerne les performances des indicateurs de la surveillance, 4 indicateurs n'ont pas atteint les performances escomptées: Le pourcentage des échantillons arrivés au laboratoire national moins de 3 jours après les prélèvements est resté en deçà des objectifs, variant entre 40% et 70%. Il en est de même des Districts de santé ayant notifies au moins un cas (50 à 68%). Le pourcentage des prélèvements dans lesquels un entérovirus non polio a été isolé a connu une évolution en dents de scie avec des valeurs de 0.0% en 2008 et en 2010 et 1.9% en 2011. L'examen de suivi du60emejour n'a concerné que les cas dont l’échantillon était jugé inadéquat au laboratoire.

**Conclusion:**

Les PFA qui ont été notifiés en Mauritanie de 2008 à 2012 présentent tableau clinique qui est classique, et sont notifiés avec un pic régulier en début des périodes de soudure (Mars-Juillet) suggérant l'hypothèse d'un lien écologique avec les changements de climat et de régime alimentaire. Les performances des indicateursde la surveillance des PFA sont à améliorer, notamment: 1) La compréhension des critères de jugement de la qualité des échantillons. 2) L'examen de laboratoire et l'interprétation des résultats. 3) La détermination du diagnostic final pour chaque cas de PFA non polio. 4) L'enregistrement des informations dans la base des données.

## Introduction

La Mauritanie avait soutenu sa documentation nationale pour la certification de l’éradication de la poliomyélite à la revue annuelle générale de la Commission Régionale de Certification de l'Eradication de la poliomyélite pour l'Afrique(CRCA) tenue à Brazzaville du 8 au 10 Octobre 2007 et elle avait été acceptée [1]. Dès lors, Le pays était déclaré « libéré de la poliomyélite ». Cependant, pour que cette documentation soit acceptée, il faut qu'elle réponde à un certain nombre de critères parmi lesquels une surveillance adéquate des PFA. Pour rappel, la poliomyélite est une maladie virale très contagieuse due à l'infection par le virus sauvage des types sérologiques 1, 2 et 3. Elle peut atteindre tout le monde, mais la cible principale est constituée des enfants de moins de 5 ans. Chez 0,5% à 1% des enfants infectés, le virus migre vers le système nerveux central et provoque une faiblesse musculaire et des paralysies irréversibles, principalement des membres inférieurs. Ces paralysies peuvent atteindre les muscles diaphragmatiques, provoquer des troubles respiratoires et voire la mort. La paralysie flasque qui en résulte est semblable à celle qu'on retrouve dans d'autres syndromes cliniques bien connus tels que le syndrome de Guillain barré, les myélites transverses, les névrites traumatiques, les autres entérovirus non polio, et certaines toxines (morsure de serpent, toxines, métabolique, médicament etc…) [2]. La surveillance par un réseau de laboratoires capables de déterminer l'origine virale des cas de PFA est l'une des 4 stratégies recommandées par l'OMS pour l’éradication de la poliomyélite [3]. C'est la qualité de cette surveillance qui permet de savoir si le pays est capable de détecter un PVS en circulation. La surveillance permet ainsi de contenir le PVS dans ses derniers retranchements qui sont aujourd'hui le Nigeria, le Pakistan et l'Afghanistan [4], en détectant précocement les éventuels cas d'importations capables d'anéantir un travail commencé depuis bientôt un quart de siècle lorsqu'en 1988 l'initiative pour éradiquer la poliomyélite était lancée à la 41eme Assemblée Mondiale de la Santé.[5]Bien que la PFA soit le signe le plus visible de l'infection, celle-ci n'apparaît que dans de rares cas à raison de 1/200 pour le virus sauvage type 1, 1/1200 pour le virus sauvage type 3 et 1/2000 pour le virus sauvage type 2 [6]. Ainsi, une transmission silencieuse et rapide s'opère longtemps avant l'apparition du premier cas. C'est pour cette raison que l'OMS considère tout cas confirmé de paralysie poliomyélitique comme une épidémie. L'infection est essentiellement interhumaine et n'est pas curable, mais une vaccination efficace à l'aide de 4 doses du vaccin orale confère l'immunité à vie [7]. L’éradication de la maladie par le vaccin est le principal enjeu du siècle en cours et le bout du tunnel n'a jamais été aussi proche. Les 4 stratégies recommandées par l'OMS durant la période de cette étude et qui étaient mises en œuvre pour cette fin sont: atteindre une couverture de routine élevée à au moins 95% avec 4 doses du vaccin orale dans la première année de vie; organiser les activités de vaccination de masse contre la poliomyélite pour atteindre tous les enfants de 0 à 5ans; organiser les campagnes de vaccination au porte-à-porte pour le ratissage des zones à haut risque de transmission; la surveillance à travers la détection précoce et l'analyse au laboratoire des prélèvements de tous les cas de PFA chez les enfants de moins de 15 ans.

La surveillance renforcée des PFA est mise en place en Mauritanie depuis 1998 et a permis d'atteindre le statut de « polio free » en 2007. Cependant, elle doit rester une forteresse anti- polio jusqu´à la confirmation du statut « world polio free » en maintenant un niveau de performance capable d'identifier et d'investiguer tous les cas de PFA. Les aspects caractéristiques de la paralysie flasque aiguë due à la poliomyélite sont entre autres, la fièvre au début des symptômes, la progression rapide et asymétrique de la paralysie, l'existence d'une paralysie résiduelle après 60 jours, la préservation des fonctions sensorielles. Cette étude se propose de: décrire les aspects épidémiologiques des PFA enregistrés en Mauritanie de 2008 à 2012; évaluer les performances de la surveillance durant une période de 5 ans après la certification « polio free » de 2007; identifier les composantes à améliorer dans le système de surveillance des PFA en Mauritanie.

## Méthodes

### Caractéristiques du pays et type d’étude

La Mauritanie est un vaste pays saharien situé sur la côte Nord-ouest du continent Africain, d'une superficie de 1030700km2et situé entre le 15eme et le 17emeparallèle Nord. Elle est limitrophe de la Côte Atlantique à l'Ouest, de l'Algérie et du Sahara Occidental au Nord, du Mali à l'Est et du Sénégal au Sud. En 2012, la population générale est estimée à environ 3307697 habitants sur la base du recensement effectué par l'office national de la statistique en 2001 et assumant une croissance constante à 1.024 par an [8]. Cette population est extrêmement dispersée, mais aussi très inégalement distribuée dans les 13 régions ou Wilayas que compte le pays. Plus d'un quart de cette population se trouve dans la capitale Nouakchott et on observe une légère densification des peuples dans les régions du sud entre 10 et 20habitants/km2. Les wilayas du Nord en zones plus arides sont très faiblement peuplées avec des densités inférieures à 1habitant au km2. La population de moins de 15 ans est estimée à 1620771 soit 49% de la population générale. Cette étude est une analyse rétrospective des cas de PFA survenus dans cette population de moins de 15 ans et notifiés de 2008 à 2012 à partir des données du service de surveillance épidémiologique du Ministère de la Santé Publique.

### Organisation du système de surveillance des PFA

Le système de surveillance des PFA a été mis en place en Mauritanie à partir de 1998 et s'est progressivement consolidé à la faveur de l'effort global pour l’éradication de la poliomyélite. Les activités de surveillance des PFA sont intégrées aux activités de surveillance des autres maladies épidémiques et sont mises en œuvre à tous les trois niveaux de la pyramide sanitaire. Elles sont mises en œuvre de façon passive et/ou active, à la fois dans les structures sanitaires publiques et privées et dans la communauté. La surveillance passive se déroule lors des consultations courantes des malades dans les formations sanitaires tandis que pour la surveillance active, les visites des sites de surveillance sont effectuées périodiquement pour faire une revue des registres de consultation et d'hospitalisation en vue de rechercher les cas qui seraient passés inaperçus. Il existe au total 580 sites de surveillance dans le pays parmilesquels25 sites de haute priorité qui sont visités de façon hebdomadaire, 67 sites de moyenne priorité visités de façon bimensuelle et 488 sites de faibles priorités visités mensuellement.

### Niveau périphérique

Les chefs de poste de santé associent les membres de la communauté et les tradi-praticiens à la surveillance de l'aire de santé. Dans sa forme passive, ils identifient les cas de PFA au cours des consultations courantes, notifient au Chef de District de Santé(Moughataa) et procèdent à l'investigation et aux prélèvements de 2 échantillons avant le 14eme jour du début de la paralysie et ce dans un intervalle maximum de 24heures.

Les chefs de District de santé et leurs assistants en surveillance sont bien formés et assurent la formation continue des chefs de poste de santé, des membres de la communauté, des tradi-praticiens et des praticiens des autres médecines parallèles. Ils notifient également le niveau régional, participent également à l'investigation et au prélèvement des échantillons de selles ainsi qu’à l'examen du 60eme jour. Ils assurent la compilation des données et les transmet mensuellement agrégées au niveau Régional.

### Niveau Régional

Au niveau régional(Wilaya), il existe un point focal régional qui est comptable des activités de surveillance des PFA dans la région. Il participe aux réunions périodiques au niveau central; il effectue les visites des sites de surveillance au niveau périphérique pour la surveillance active dans les hôpitaux et la formation des personnels impliqués dans le système;il participe également à l'investigation et au prélèvement des échantillons de selles et à l'examen du 60eme jour des cas de PFA. Il assure la sensibilisation auprès des autres prestataires parallèles ainsi que le plaidoyer auprès des autorités administratives.

### Niveau National

Le niveau national élabore et diffuse les supports de surveillance des PFA, collecte les données, analyse et diffuse à tous les niveaux, assure la disponibilité des kits de prélèvement au niveau de tous les sites de surveillance, collecte les échantillons, prépare et envoie au laboratoire de référence à Dakar, participe à l'investigation des cas suspects des grands centres hospitaliers publics et privés de la capitale. Dans les établissements publics et privés de la ville de Nouakchott, les points focaux de surveillance des PFA sont chargés d'alerter lorsqu'un cas suspect est identifié.

### Indicateurs de performance de la surveillance des PFA

L'analyse de la performance de la surveillance des PFA est faite à l'aide des indicateurs standards dont les objectifs ont été fixés par l'OMS. Les indicateurs ci-dessous ont été analysés.

**Indicateur 1:** Le taux de PFA chez les enfants de moins de 15 ans. C'est le nombre de cas de PFA enregistrés pour 100000 enfants de moins de 15 ans. (Objectif OMS > 2);

**Indicateur 2:** Le pourcentage des cas de PFA pour lesquels 2 prélèvements sont recueillis dans les 14 jours (objectif OMS>80%);

**Indicateur 3:** Le pourcentage de cas de PFA pour lesquels un suivi à 60 jours est effectué pour vérifier si le patient présente une paralysie résiduelle (objectif OMS>80%);

**Indicateur 4:** Le pourcentage des prélèvements arrivés au laboratoire moins de 3 jours après leur envoi (Objectif OMS>80%);

**Indicateur 5:** Le pourcentage des prélèvements de selles arrivés au laboratoire dans de bonnes conditions (température < 8°c, volume des selles, absence de dessiccation) (Objectif OMS>80%);

**Indicateur 6:** Pourcentage des prélèvements pour lesquels les résultats sont envoyés dans les 28 jours après leur réception au laboratoire (objectif OMS > 80%);

**Indicateur 7:** Pourcentage des prélèvements pour lesquels un entérovirus non poliovirus est isolé (objectif OMS>10%);

**Indicateur 8:** Pourcentage des cas investigués dans les 48heures après la notification (Objectif OMS>80%).

### Analyse des données

Les données ont été analysées à l'aide du logiciel Epi info version 3.4.3 (CDC Atlanta). Cette analyse a permis de décrire la présentation épidémiologique des cas de PFA et d’évaluer les indicateurs de la performance du système de surveillance. Les graphiques ont été faites à l'aide d'Excel 2007.

## Résultats

**Figure 1:**
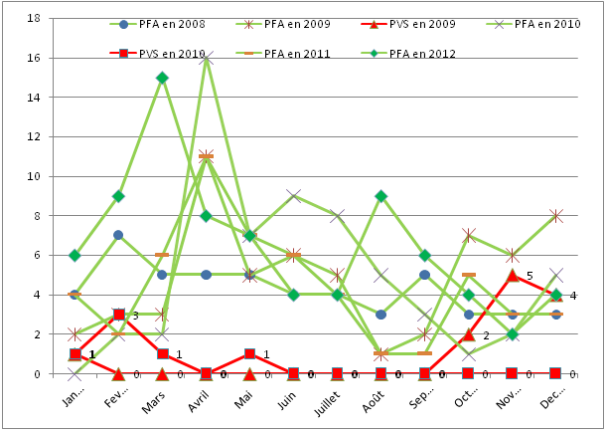
Incidence mensuelle des PFA notifiés en Mauritanie, 2008-2012

Epidémiologie: Le nombre de cas de PFA rapportés durant la période a été de 319 soit une incidence moyenne de 4.61/100000 enfants de moins de 15 ans par an. L’âge moyen était de 4 ans (E.T. ±4ans). 77.4% des cas avaient l’âge inférieur ou égal à 5ans. 5.3% avaient un âge supérieur à 10ans et 17.3% un âge supérieur à 5 ans et inférieur ou égal à 10ans. Le ratio sexe était de 1.21 (1.21 pour les PFA non polio et 1.25 pour les PFA confirmés PVS) en faveur des garçons (Tableau 1).18 cas de PFA ont été confirmés poliovirus sauvage dont 6 en 2009 et 12 en 2010 et tous d'importation. L’âge moyen était de 3.4 ans (E.T ±2.6 ans). 44,4%étaient des filles et 55.5% garçons. Cette proportion était proche de celle des PFA non polio (45.1% versus 54.9%) (Tableau 1). Les cas de PFA étaient classés en fonction du nombre de doses du vaccin orale reçues, 61% avaient reçu au plus une dose de VPO pour les cas de PFA confirmés polio contre 7.4% pour les PFA non polio. Aucune discrimination de genre n'a été observé sur la population des PFA ayant reçu une dose au plus (ratio-sexe = 16/17 = 0.94) (Tableau 2). La distribution des cas cumulés par mois montre une importante notification des cas PFA pendant la période de Février à juillet alors que l'incidence des cas confirmés a été plus importante entre Novembre et Février. Cependant, on note une importante notification qui a suivi le déclenchement de l’épidémie en 2009 (Figure 1). Après investigation de l'ensemble des cas de PFA qui ont été notifiés, la fièvre était présente pour 272 cas des PFA non polio soit 90% tant dis que ce pourcentage était de 85% pour les cas PVS. Cette fièvre à progresser en 3 jours pour tous les cas de PVS et pour 82,7% des cas de PFA non polio. Le taux d'hospitalisation était de 13.6% pour les cas de PFA non polio contre 89% pour les PFA qui ont été confirmés PVS. Dans tous les deux groupes, les membres de prédilection étaient d'abord l'un des 2 membres inférieurs de façon alternative (46.8%), ensuite les deux membres inférieurs à la fois (22.2%). En troisième lieu, un membre supérieur et un membre inférieur de façon asymétrique (5.3%) et en quatrième lieu, tous les 4 membres (4.6%). (Tableau 3). Le diagnostic final des cas de PFA non polio n’était pas rapporté pendant toute la période de l’étude, cependant, 2 cas ont été classés compatible et un cas de paralysie associée à la vaccination contre la polio a été rapporté en 2012 chez un enfant féminin de 3ans ayant reçu sa première dose de VPO. Cette paralysie a évolué de façon progressive sur les deux membres inférieurs et était associée à la fièvre.

**Tableau 1:** Fréquence des PFA par groupe d’âges et par sexe

Age	<1ans		1-5ans		6-10ans		>10ans		Total	
Sexe	Garçon	Fille	Garçon	Fille	Garçon	Fille	Garçon	Fille	Garçon	Fille
**PFA non polio**	46	31	81	74	31	21	7	10	165	136
**PFA polio**	2	3	5	5	3	0	0	0	10	8
**Total sexe spécifique**	48	34	86	79	34	21	7	10	175	144
**Total**	82		165		55		17		319	
**Pourcentage**	25.7%		51.7%		17.2%		5.3%		100%	

**Tableau 2:** Fréquence de PFA par nombre de doses de VPO reçu et par sexe

Nombre de doses de VPO reçu	Nombre de cas de PFA non polio					Nombre de cas de PFA polio confirmé				
	Garçon	Fille	Total	%	%cumule	Garçon	Fille	Total	%	%cumulé
**0**	5	6	11	3.7%	3.7%	5	1	6	33.3%	33.3%
**1**	3	8	11	3.7%	7.4%	3	2	5	27.8%	61.1%
**2**	12	8	20	6.7%	14.1%	0	2	2	11.1%	72.2%
**3 +**	149	110	259	85.9%	100%	2	3	5	27.8%	100%

**Tableau 3:** Caractéristiques cliniques des cas de PFA

	PFA non polio (n = 301)			PFA polio (n = 18)		
PRESENTATION CLINIQUE						
	oui	non	Non précisé	oui	non	Non précisé
Fièvre au début	272	27	1	16	2	1
Progression en 3jours	249	52	0	16	1	1
Paralysie asymétrique	160	141	0	7	11	0
PFA de début brutal	275	26	0	15	3	0
Hospitalisation	36	265	0	8	9	1
**LOCALISATION DE LA PARALYSIE**						
Les 2 membres inferieurs	84			9		
Membre inferieur gauche	74			3		
Membre inférieur droit	67			3		
Membre supérieur gauche et membre inférieur droit	24			0		
Membre supérieur droit et Membre inférieur gauche	16			1		
Tous les 4 membres	14			1		
Membre supérieur gauche	5			0		
Membre supérieur droit	4			1		
Les 2 membres inférieurs et le membre supérieur gauche	4			0		
Les 2 membres supérieurs	2			0		
Les 2 membres du côté gauche	2			0		
Les 2 membres inférieurs et le membre supérieur droit	2			0		
Les 2membres du côté droit	0			0		
Les 2 membres supérieurs et le membre inférieur droit	1			0		
Localisation non précisée	2			0		
**RESULTAT DE LA CULTURE CELLULAIRE**						
Négatif	266			0		
Entérovirus non polio	17			0		
Suspect Polio	12			12		
Suspect Polio &entérovirus non polio	5			6		
Non précisé	1			0		

Performances de la surveillance: 4 indicateurs n'ont pas atteint les performances escomptées: Le pourcentage des échantillons arrivés au laboratoire national moins de 3 jours après les prélèvements est resté en deçà des objectifs, variant entre 40% et 70%. IL en est de même des Districts de santé ayant notifies au moins un cas (50 à 68%). Le pourcentage des prélèvements dans lesquels un entérovirus non polio a été isolé a connu une évolution en dents de scie avec des valeurs de 0.0% en 2008 et en 2010 et 1.9% en 2011, mais supérieur à10% en 2009 et 2012. L'examen de suivi du60emejour n'a concerné que les cas dont l’échantillon était jugé inadéquat au laboratoire (Tableau 4).

**Tableau 4:** Les indicateurs des performances de la surveillance des PFA en Mauritanie, 2008-2012

Indicateurs	Objectif OMS	2012	2011	2010	2009	2008
Population cible estimée (<15ans)		1620771	1582784	1545688	1509461	1474083
Nombre de cas de PFA notifiés		78	53	66	71	51
Nombre de cas de PVS confirmés		0	0	6	12	0
Taux de PFA non polio chez les enfants de moins de 15 ans	≥2/100.000	4.8	3.3	4.2	4.7	3.4
Pourcentage des cas de PFA pour lesquels 2 prélèvements de selles sont recueillis dans les 14 jours	>ou = 80%	96%	85%	98%	97%	100%
Pourcentage de cas de PFA pour lesquels un suivi à 60 jours est effectué pour vérifier si le patient présente une paralysie résiduelle.	>ou = 80%	2.5%	1.9%	4.5%	14.5%	5.5%
Pourcentage de prélèvements arrivés au laboratoire moins de 3jours après leur envoi	>ou = 80%	51.3%	52.8%	72.7%	40.8%	60.8%
Pourcentage de prélèvements de selles arrivés au laboratoire dans de bonnes conditions (température < 8°c, 7-10g volume, absence dessiccation.	>ou = 80%	97.43%	98.1%	95.45%	85.5%	94.11%
Pourcentage de prélèvements pour lesquels les résultats sont envoyés au district dans les 28 jours après leur réception au laboratoire	>ou = 80%	100%	100%	100%	98.6%	100%
Pourcentage de prélèvements pour lesquels un entérovirus non polio (NPENT) a été isolé.	>ou = 10%	16.7%	1.9%	0.0%	11.26%	0.0%
Pourcentage des districts de santé ayant notifiés au moins un cas	>ou = 80%	67.9%	49.05%	64.15%	58.5%	49.05%
Pourcentage des cas investigués dans les 48heures après la notification	>ou = 80%	96.2%	98.1%	94%	97.2%	98%
pourcentage des PFA non polio n'ayant jamais reçu de vaccin VPO	0.0%	2/78= 2.56%	2/42= 7.14%	9/60= 15%	3/71= 4.22%	1/51= 1.96%

## Discussion

**Figure 2:**
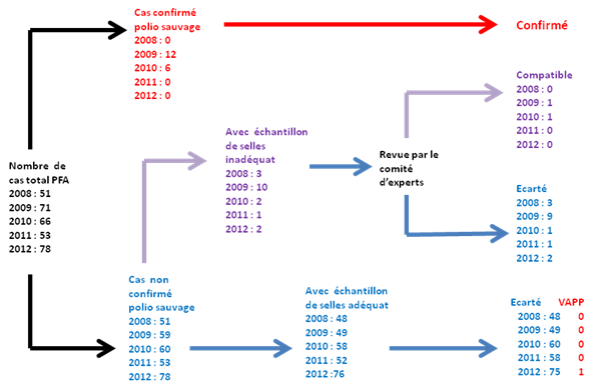
Diagramme de flux montrant la classification virologique des cas de PFA en Mauritanie

La surveillance des PFA est le « Gold standard » pour le contrôle de la circulation du PVS et, pour cette raison, elle doit être précise à la fois dans la continuité de toutes les démarches de détection, d'investigation et de suivi, mais aussi d'enregistrement et de sécurisation des informations et des données qui en découlent. Dans le meilleur des cas,et c'est souvent ainsi dans les pays assez développés, chaque cas de PFA doit avoir son diagnostic précis au terme d'une investigation clinique approfondie, accompagnée d'une batterie d'examens des plus simples au plus sophistiqués en fonction de l’étiologie recherchée. Dans le moindre des cas, à chaque cas de PFA doit correspondre un diagnostic le plus probable! Le comité d'experts polio a été recommandé et installé dans les pays pour d'une part, parvenir à cette fin. La plupart des pays aujourd'hui indemnes de polio virus sauvage sont parvenus au moins à déterminer le diagnostic le plus probable de chaque cas de PFA pendant la phase terminale de la libération. En dehors des 18 cas qui ont été confirmés poliovirus sauvage dont 6 en 2009 et 12 en 2010, aucun diagnostic final n'a été rapporté pour les autres 301 cas de PFA notifiés en Mauritanie depuis la certification « polio free » de 2007. L'examen de suivi pratiqué à partir du 60eme jour du début de la paralysie à la recherche d'une paralysie résiduelle permet au comité d'experts de parvenir à la classification finale des cas, et éventuellement, parvenir à un diagnostic certain ou au diagnostic le plus probable. Pendant la période de l’étude, cet examen de suivi était restreint seulement au cas dont les échantillons étaient jugés inadéquats au laboratoire dans le but de classifier sans pour autant parvenir à un diagnostic final. Par conséquent, la prévalence des étiologies liées aux PFA en Mauritanie est méconnue. Cependant, comparativement à la plupart des études, 77.4% ont un âge inférieur à 5ans contre, 82.5% à akwa ibom (au Nigeria) [9], 90% en Inde [10], 74.3% à Ibadan ( au Nigeria) [11] 37% à Marches (Italy) [12], 40% à Hamadan en Iran [13]. La fièvre est présente pour 21.6% dans une série de 88 cas à hamadan à l'Ouest de l'Iran [13]. 84% ont une progression complète en 4 jours contre 82% en 3 jours dans notre série [13]. Il n'y a pas une prédilection du genre quelque soit le groupe d’âge. On constate que les PFA sont notifiés intensément au début de l'année avec un pic régulier dès la fin du mois de Février jusqu´à la fin du mois de Juillet. Cette période correspond aussi à la période de soudure en Mauritanie, ce qui permet d'envisager l'hypothèse d'un lien écologique avec un changement brusque de climat et de régime alimentaire. L'hypothèse alternative étant peut être une notification en excès en début d'année dans le but d'atteindre le taux minimal de l'indicateur de référence qui est de 2cas de PFA pour 100000 enfants de moins de 15 ans par année et un relâchement lorsque l'indicateur est déjà atteint. Cette dernière hypothèse est renforcée par le fait que les pics de notification des PFA ont été très importants les mois qui ont suivi les épidémies de 2009 et 2010. (Figure 2). Durant toute la période 30 à 50% de Districts de santé sont silencieux chaque année ce qui pourrai bien représenter une menace pour la capacité de détection d'un PVS par le système de surveillance. La qualité des prélèvements des selles qui arrivent au laboratoire est déterminante pour le succès de la surveillance de PFA car de cette qualité découle la capacité de détecté un virus s'il existe. Bien que cet indicateur soit resté au-dessus des 80% tel que recommandé par l'OMS, non seulement les délais d'acheminement au laboratoire n'ont pas été satisfaisants (40 à 70%) mais aussi, cela ne s'est pas traduite au niveau des résultats des investigations en 2008, 2009 et 2010 en ce qui concerne le pourcentage des entérovirus non polio dans les selles qui doit être supérieur à 10%. Ceci pourrait se justifier soit par des limites dans l'appréciation de la qualité des échantillons de selles soit dans le report des résultats des investigations des échantillons dans la banque des données.

## Conclusion

Les PFA qui ont été notifiés en Mauritanie de 2008 à 2012 présentent tableau clinique qui est classique, et sont notifiés avec un pic régulier en début des périodes de soudure (Février-Juillet) suggérant l'hypothèse d'un lien écologique avec les changements de climat et de régime alimentaire. Les performances des indicateurs de la surveillance des PFA sont à améliorer, notamment: 1) La compréhension des critères de jugement de la qualité des échantillons. 2) L'examen de laboratoire et l'interprétation des résultats. 3) La détermination du diagnostic final pour chaque cas de PFA non polio. 4) L'enregistrement des informations dans la base des données.
